# Pulmonary Hypertension and Hyperglycemia—Not a Sweet Combination

**DOI:** 10.3390/diagnostics14111119

**Published:** 2024-05-28

**Authors:** Or Bruck, L. M. Pandit

**Affiliations:** 1 Section of Pulmonary, Critical Care, Sleep Medicine, Baylor College of Medicine, Houston, TX 77024, USA; or.bruck@bcm.edu; 2 Michael E. DeBakey Veterans Affairs Medical Center, Center for Translational Research on Inflammatory Diseases (CTRID), Houston, TX 77030, USA

**Keywords:** vascular inflammation, endothelial dysfunction

## Abstract

Hyperglycemia and pulmonary hypertension (PH) share common pathological pathways that lead to vascular dysfunction and resultant cardiovascular complications. These shared pathologic pathways involve endothelial dysfunction, inflammation, oxidative stress, and hormonal imbalances. Individuals with hyperglycemia or pulmonary hypertension also possess shared clinical factors that contribute to increased morbidity from both diseases. This review aims to explore the relationship between PH and hyperglycemia, highlighting the mechanisms underlying their association and discussing the clinical implications. Understanding these common pathologic and clinical factors will enable early detection for those at-risk for complications from both diseases, paving the way for improved research and targeted therapeutics.

## 1. Introduction

Pulmonary hypertension is a progressive vasculopathy involving the expanse of the pulmonary vascular bed characterized by endothelial cell dysfunction and smooth muscle cell proliferative hypertrophy among its various defining histopathologic attributes [1]. The pathologically increased pulmonary vascular resistance that occurs secondary to this vasculopathy diminishes pulmonary blood flow and gas exchange, and leads to progressive right heart failure and associated increased mortality. Pulmonary hypertension is classified into five separate groups, broadly separated by associated and causative clinical characteristics [2,3]. While alterations in metabolism and glycemic homeostasis have been primarily described in human and animal models of pulmonary arterial hypertension (Group 1—PAH) [4,5,6,7], metabolic alterations can exist across other PH groups [6]. Altered glucose metabolism is associated with cardiovascular and chronic lung diseases that are implicated in the development of other types of pulmonary hypertension (Groups 2, and 3, respectively). Metabolic syndrome and pulmonary hypertension, though traditionally viewed as distinct entities, exhibit compelling associations that extend beyond shared comorbidity, including shared pathophysiological mechanisms, clinical implications with synergistic outcomes, and potential therapeutic interventions. Pulmonary hypertension (PH) and insulin resistance reveal overlapping molecular mechanisms, suggesting an interplay between these diseases. Specific signaling cascades, including dysregulation of the PI3K/Akt pathway, inflammation, and endothelial dysfunction, emerge as common factors, suggesting a potential bidirectional influence between insulin resistance and PH. Chronic inflammation, oxidative stress, hormonal factors and endothelial dysfunction—common features in metabolic disorders—are also implicated in the pathogenesis of pulmonary hypertension [4,5,6]. Epidemiological and clinical studies establish a strong correlation between metabolic syndrome components (obesity, dyslipidemia, insulin resistance, and hypertension) and the incidence and severity of pulmonary hypertension [8]. Research suggests patients with pulmonary hypertension are at a higher risk for complications from the insulin resistance and concomitant impaired glucose metabolism and insulin resistance can exacerbate the already compromised cardiovascular function in pulmonary hypertension, leading to an increased risk of adverse outcomes such as progressive vascular remodeling, right heart failure and overall decreased functional capacity [8]. Understanding the interplay between pulmonary hypertension and the insulin resistance that characterizes metabolic syndrome is crucial for comprehensive patient care and may pave the way for targeted interventions to improve clinical and quality of life outcomes.

## 2. Pathophysiology of Pulmonary Hypertension

**Figure 1:**
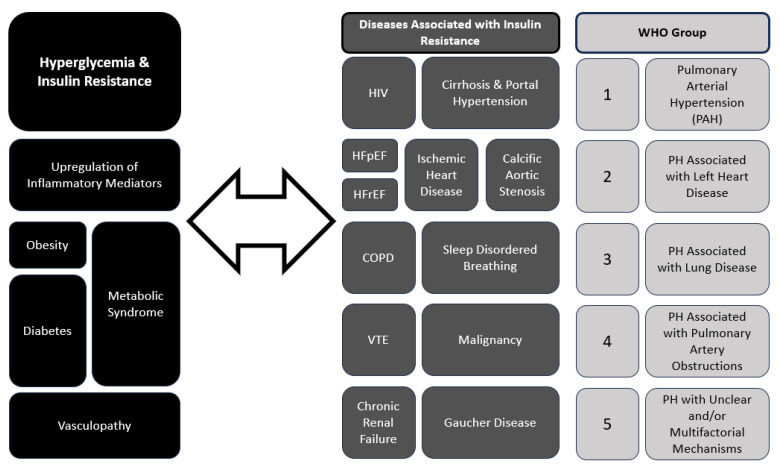
Interplay between Hyperglycemia, Insulin Resistance and Pulmonary Hypertension (PH). HIV—human immunodeficiency virus; HFpEF—heart failure with preserved ejection fraction; HFrEF—heart failure with reduced ejection fraction; COPD—chronic obstructive pulmonary disease; VTE—venous thromboembolic disease.

Pulmonary hypertension can arise from various etiologies, including pulmonary arterial hypertension (PAH), pulmonary hypertension due to left heart disease (PH-LHD), pulmonary hypertension associated with chronic lung disease, and chronic thromboembolic pulmonary hypertension (CTEPH). Current guidelines describe five groups of PH designated by the World Health Organization (WHO Groups 1–5) with shared pathophysiological and clinical features (See Figure 1, WHO groups) [1,3,9,10]. Within the pulmonary vascular bed, all compartments, namely PAs, arterioles, capillaries, veins and venules, are involved in the remodeling process to different degrees, depending on the underlying associated systemic conditions and clinical subgroup [1,10]. In all of groups of PH, the underlying vascular remodeling leads to shared hemodynamic alterations of increased pulmonary vascular resistance (PVR) and resultant increased right ventricular pressure overload and heart failure. In PAH (Group 1), vascular remodeling is characterized by endothelial dysfunction, and vasoconstriction contributing to elevated pulmonary vascular resistance (PVR) [10]. Similarly, in CTEPH (Group 4), parallel processes of (i.) trans endothelial migration, (ii.) heightened expression of endothelial adhesion molecules, and (iii.) dysfunctional endothelial tissue are upregulated with an overall influx of inflammatory cells that characterize chronic venous thromboembolism leading to increased PVR [9]. The pathophysiology of PH-LHD (Group 2) involves left ventricular dysfunction and elevated left-sided filling pressures, which subsequently lead to chronically elevated pulmonary vascular bed pressures and remodeling. Chronic lung disease-associated PH (Group 3) is at least in part due to chronic hypoxemia or in the case of obstructive sleep apnea chronic intermittent nocturnal hypoxemia, leading to unopposed hypoxia-induced vasoconstriction and increased PVR. In all PH clinical groups, the concomitant presence of PH worsens morbidity of the underlying associated conditions and leads increased mortality, usually as a complication of right-sided heart failure [1,2]. 

## 3. The Interplay between Pulmonary Hypertension and Hyperglycemia

Recent studies have suggested a bidirectional relationship between PH and hyperglycemia [4,8,11]. When a diagnosis of PAH is made in elderly patients who present with cardiovascular comorbidities such as diabetes mellitus and obesity, early identification and treatment of PAH can improve outcomes. Endothelial dysfunction, a common denominator in both conditions, plays a pivotal role in their pathogenesis [1,11,12]. Endothelial injury and inflammation contribute to vascular remodeling and vasoconstriction in PH, and decreased endothelium-dependent vasodilation and hyperpermeability, is the initial stage of vasculopathy. Endothelial injury can serve as a vital prognostic indicator of diabetic complications [12] and a signal for impaired glucose metabolism and insulin signaling. The association between PH and hyperglycemia has significant clinical implications. Recent and ongoing analyses of a large European PAH database have revealed that patients with PAH and cardiovascular comorbidities such as diabetes benefit from PAH and diabetes treatment, with improvements in functional status, serologic biomarkers, and mortality risk [13]. Patients with PH, particularly those with PAH, are at increased risk of insulin resistance and DM. Conversely, individuals with DM have a higher prevalence of PH, contributing to worse outcomes and increased mortality [13]. Screening for PH in diabetic patients with unexplained dyspnea or exercise intolerance and evaluating for DM in those with PH may aid in early detection and intervention. Furthermore, optimizing glycemic control and addressing shared comorbidities such as obesity and obstructive sleep apnea are crucial in the management of both conditions. Figure 1 summarizes the interplay between the different subgroups of PH and diabetes mellitus (DM) and hyperglycemia.

## 4. PH and Diabetes Mellitus Have Shared Pathophysiological Mechanisms

Both DM and PH are characterized by endothelial dysfunction, marked by impaired nitric oxide bioavailability, increased oxidative stress, and inflammation [1,11,12]. Endothelial dysfunction contributes to vasoconstriction, vascular remodeling, and thrombosis in the pulmonary vasculature, exacerbating PH vasculopathy. Chronic low-grade inflammation, observed in both DM and PAH, plays a pivotal role in the pathogenesis of vascular dysfunction and remodeling. Inflammatory mediators such as cytokines, chemokines, and growth factors contribute to endothelial injury, smooth muscle cell proliferation, and fibrosis in the pulmonary vasculature [1,11]. Several common inflammatory mediators play pivotal roles in both pulmonary hypertension (PH) and metabolic pathways, contributing to the interconnection between impaired glucose metabolism and PH [11,12]. Some of the key inflammatory mediators (see Table 1) involved include:

### 4.1. Tumor Necrosis Factor-alpha (TNF-α)

#### 4.1.1. Role in Insulin Resistance

The cytokine tumor necrosis factor-α (TNF-α), and its receptors, 1 and 2 (TNFR1, TNFR2), are members of TNFR superfamily, and are important mediators of chronic inflammation in diabetes mellitus [14,15]. Elevated levels of TNF-α have been associated with impaired glucose uptake and decreased insulin sensitivity in various tissues [14]. Experimental studies have shown that TNF-α activation is involved in chronic inflammatory reactions within the kidney with measured increased serum levels in diabetic complications, particularly diabetic nephropathy [15]. Elevated TNF-α leads to the development of glomerulosclerosis and tubulointerstitial fibrosis, and is implicated in the progressive renal dysfunction in diabetes. 

#### 4.1.2. Role in PH

NF-α is also implicated in the inflammation and endothelial dysfunction seen in pulmonary hypertension, contributing to vascular remodeling and increased pulmonary vascular resistance [16]. Specifically, during hypoxia or tissue ischemia, TNF-α activates and promotes neutrophil aggregation, and induces adhesion molecule expression, resulting in microvascular obstruction. These cascading events lead to vascular endothelial damage. In animal high-altitude models of PAH, when rodents were exposed to incremental increases in exposure time to high-altitude at 10, 20, and 30 days, measured TNF-α levels in pulmonary arteries of these high-altitude exposed rodents were also proportionately increased with increased exposure, whereas TNF-α levels in rodents reacclimated to low altitude were measurably reduced [17].

### 4.2. Interleukin-6 (IL-6)

#### 4.2.1. Role in Insulin Resistance

IL (interleukin)-6 has been linked to insulin resistance, particularly in obesity-related metabolic disorders, interfering with insulin signaling and contributing to systemic inflammation [18,19,20]. Genetic knockout of IL-6 inflammatory pathways ameliorates the effects glucose intolerance and insulin insensitivity in an animal model of high fat induced metabolic syndrome [19].

#### 4.2.2. Role in PH

Previous studies have shown that IL-6 is elevated in PAH and independently associated with indices of right ventricular function [21]. IL-6 contributes to vascular remodeling and inflammation in pulmonary arteries [21,22]. Across human tissue sources, the lung has the second highest expression of IL-6 at the RNA level with measurable upregulated membrane-bound IL-6 receptor in pulmonary artery smooth muscle cells (PASMCs) in patients with idiopathic PAH (IPAH) [21,23,24]. However, prior investigations of IL-6 as a prognostic biomarker of mortality have been inconsistent in the small (PAH) cohorts [22]. Larger analyses are needed to clarify whether IL-6 is a marker of critical illness or a mechanistic biomarker of pulmonary vascular remodeling.

### 4.3. C-Reactive Protein (CRP)

#### 4.3.1. Role in Insulin Resistance

Elevated CRP levels are associated with insulin resistance, and are considered a marker of systemic inflammation in metabolic disorders [25].

#### 4.3.2. Role in PH

CRP levels are often increased in pulmonary hypertension and are indicative of inflammation. CRP is associated with endothelial dysfunction, and may contribute to the progression of pulmonary vascular remodeling.

### 4.4. Nuclear Factor-kappa B (NF-κB)

#### 4.4.1. Role in Insulin Resistance

NF-κB is a key regulator of inflammatory responses, and is implicated in insulin resistance by promoting the expression of pro-inflammatory cytokines [26]. Several animal models of metabolic disease have also demonstrated that inhibition of NF-κB pathway-related metabolic inflammation attenuates obesity-associated insulin resistance and hepatic steatosis [27].

#### 4.4.2. Role in PH

Transcription factor NF-κB activation plays essential roles in multiple pathological processes, with its signaling pathway activated by hypoxia, a known stimulus for pulmonary vasoconstriction; with unopposed chronic hypoxic exposure, pulmonary hypertension results [28]. NF-κB activation is observed in both human and animal models of pulmonary hypertension and contributes to vascular inflammation, endothelial dysfunction, and smooth muscle cell proliferation [29].

### 4.5. Monocyte Chemoattractant Protein-1 (MCP-1)

#### 4.5.1. Role in Insulin Resistance

Monocyte chemoattractant protein-1 (MCP-1) contributes to the development of insulin resistance in adipose tissue through its ability to promote migration and activation of proinflammatory cells. Notably, the migration of macrophages and their polarization from predominantly anti-inflammatory to proinflammatory subtype is a critical step in the development of insulin insensitivity in adipose tissue [30,31]. MCP-1 has been reported to stimulate macrophage migration, promote recruitment of immune cells to adipose tissue resulting in the observed shift to insulin resistance [30,31].

#### 4.5.2. Role in PH 

Increased MCP-1 levels are associated with animal models of pulmonary hypertension and the human PH subgroup chronic thromboembolic pulmonary hypertension (Group 4—CTEPH) Specifically, MCP-1 induces vascular remodeling by promoting inflammation and cell migration in chronic thromboemboli [32]. These chronic clots that are characteristic of CTEPH are uniquely highly adherent to the pulmonary vascular wall, and contain collagen, elastin, inflammatory cells, that differentiate them from acute fibrin laden thromboemboli.

## 5. Metabolic Dysregulation in Pulmonary Hypertension

Insulin resistance, dyslipidemia, and altered glucose metabolism are common features of both DM and PAH. Metabolic dysregulation contributes to excess cellular proliferation and mitochondrial dysfunction which promotes vascular remodeling in PAH progression. Some of the altered metabolic pathways that have been identified in PAH include abnormalities in glycolysis and glucose oxidation, fatty acid oxidation, and the tricarboxylic acid cycle. Glycolysis in particular provides a constant source of energy (ATP) in vascular endothelial cells relative to other types of cardiovascular cells [33]. Increasing evidence highlights the complexity of varied metabolic processes, in particular the reliance on glucose fermentation for growth and bioenergetics proliferation, regardless of oxygen availability (or traditionally considered “aerobic” conditions). One example of this “metabolic shift” is the Warburg effect, which describes an energy shift from mitochondrial oxidative phosphorylation to aerobic glycolysis in which pyruvate is preferentially fermented to lactate, regardless of the presence of oxygen/aerobic conditions [33,34]. The Warburg effect has been observed not only in cancer, but also in pulmonary artery endothelial cells (PAECs), which produce large amounts of lactate in their preferential glycolytic state. In PAH, glucose metabolism is preferentially diverted from mitochondrial oxidative phosphorylation towards cytoplasmic glycolysis in PAECs and pulmonary artery smooth muscle cells (PASMC). This glycolytic shift has not only been observed in primary cultured human pulmonary vascular cells, but also in cardiomyocytes derived from patients with PAH in vivo, in addition to PAH animal models [34,35]. How and “why” endothelial cells preferentially utilize glycogen and glycolysis to generate energy through a ‘Warburgesque’ phenotype is still not fully understood. Some have postulated that this glycolytic shift in PAECs allow these endothelial cells to use glucose for efficiently conferring a functional advantage over oxidative phosphorylation [33]. Moreover, endothelial cells are more resistant to hypoxia than other cell types because of preferential anaerobic glycolytic pathway particularly when glucose is readily available [33,36]. Contrastingly, when glucose levels are reduced, endothelial cells are more sensitive to hypoxic/low oxygen states, suggesting that glycolysis renders PAECs more resistant to hypoxia, and that hyperglycemia enhances this state of resistance. As such, PAH, hypoxemia and hyperglycemia are distinct clinical scenarios that inextricably bioenergetically linked. In fact, endothelial cells’ intracellular energy stores are in the form of glycogen, highlighting the importance of glycemic metabolism in endothelial cell biology [33].

Shared alterations in fatty acid oxidation are another commonality between PAH and DM, which has been observed in PAH cardiomyocytes [34,36]. Increased levels of carnitine and acylcarnitine in plasma from PAH patients, also support abnormal mitochondrial fatty acid oxidation [33]. Plasma metabolomic studies in PAH have demonstrated alterations in glycolysis, lipid metabolism, and altered bioenergetics [37]. Interestingly, these alterations in metabolism may extend to other types of PH [37,38]. In one study of chronic thromboembolic pulmonary hypertension (CTEPH) patients, the plasma metabolome of CTEPH patients revealed that plasma acylcarnitines and fatty acid and glycerol, an essential component of triglycerides and an indicator of whole body lipolysis, was 2.4 times higher in the CTEPH patients compared to healthy controls [38]. Moreover, pathway enrichment analysis identified the following pathways as enriched in the CTEPH patients compared to controls: amino sugar metabolism, acyl choline, and long-chain fatty acid oxidation [38]. To summarize, metabolic and bioenergetic alterations stemming from mitochondrial abnormalities as well as vascular cell dysfunction play a notable role I both PH and DM. Nitric oxide signaling, glucose metabolism, fatty acid oxidation, and the TCA cycle are abnormal in PAH, along with alterations in the mitochondrial membrane potential, and PAEC and vascular cellular proliferation and DM share abnormal metabolic bioenergetics, particularly when it comes to endothelial cells, that facilitate a metabolic shift in endothelial cells promoting vasculopathy in both PAH and DM [36,37].

## 6. Oxidative Stress

Oxidative stress results from an imbalance of the prooxidant–antioxidant balance of the cell, resulting in reduced antioxidant cellular activity in favor of prooxidative processes that produce excess reactive oxygen species (ROS) that are deleterious to cellular processes. Oxidative stress has been associated with a variety of pathologic conditions, including diabetes mellitus (DM) [39] and is a common feature in both pulmonary hypertension and DM, sharing a pathway to vascular dysfunction [40]. In DM, auto-oxidation of glucose, imbalanced redox reactions, decreased tissue concentrations of low molecular weight antioxidants (such as diminished Glutathione and vitamin E) are potential causes of oxidative stress and increased ROS that result in overall impaired antioxidant defense enzymes and increased DM complications [40]. The increased production of free radicals and ROS and impairment of endogenous antioxidants can impair insulin signaling pathways and contribute to insulin resistance. In the context of pulmonary hypertension, oxidative stress also contributes to vascular damage and remodeling in both human and animal models of PAH [40,41]. Vasoconstriction promoted by oxidative stress is probably one of the most critical factors in the early stages of PAH. Oxidative stress plays a key role in impairing endothelial cell function, producing an increase in the synthesis and release of endothelium-derived constrictor factors such as endothelin-1 (ET-1) and a decrease in endogenous vasodilators such as nitric oxide (NO), contributing to the alterations in the homeostasis of vascular tone and permeability [39]. Oxidative stress thus leads to a reduction in NO bioavailability when ROS, in the form of O2, reacts with NO to form peroxynitrite (ONOO−) which, in turn, reacts with available tyrosine residues of proteins producing 3-nitrotyrosine, causing lung epithelial damage [40,41]. Therefore, oxidative stress and resulting excess ROS contribute directly to pulmonary vasoconstriction and impaired regulation of pulmonary vascular tone—critical contributing mechanisms in the development of pulmonary hypertension.

## 7. Hormonal Factors

Adiponectin, an adipokine involved in glucose metabolism, is often reduced in both insulin resistance and pulmonary hypertension. Adiponectin has anti-inflammatory effects and is involved in vascular homeostasis, with decreased adiponectin levels contributing to impaired insulin sensitivity and pulmonary vascular dysfunction [42]. Elevated plasma adiponectin levels are associated with the presence of pulmonary hypertension, increased heart failure admissions and mortality risk in African Americans [43]. Another adipokine, resistin, has been associated with insulin resistance and inflammation, and may also play a role in the development and progression of pulmonary hypertension [42]. Elevated leptin levels, often seen in obesity, are associated with insulin resistance, and can also alter inflammatory and immune responses. Another example of shared hormonal factors due to obesity when considering both DM and PH is in the role of adipose tissue in exogenous estrogen production. The role of estrogen in both these diseases, particularly how it relates to adiposity, is discussed in detail later.

## 8. Genetic Susceptibility

Individuals with DM and PAH may have shared genetic factors that predispose the development of these diseases. Genome-wide association studies have implicated overlapping pathogenic pathways through identification of common genetic variants associated with both conditions. Some of these shared inherited pathways include associated nuclear and mitochondrial mutations on metabolism. Recent work in mitochondrial genetics in PAH have identified inherited biologic pathways that are preserved through maternal lineage [44]. Mitochondria genes encode oxidative phosphorylation proteins crucial for bioenergetic function that are genetically transmitted to subsequent lineages through the maternal inheritance. Mitochondrial (mt) DNA mutations occur with greater frequency compared to the nuclear genome [44,45]. Mitochondrial haplogroups are defined collections of mutations that are maternally inherited and are associated with increased or decreased risk of metabolic and autoimmune diseases, and have been associated with various types of cardiovascular disease, type 2 diabetes mellitus, and several types of cancer [46,47]. Other genetic associations between PAH and altered metabolic pathway include implications from the effects of heterozygous germline mutations of the BMPR2 gene, which is identifiable in approximately 75% of heritable (or familial) PAH and 20% of idiopathic PAH [48,49]. BMPR2 is a serine-threonine kinase receptor of transforming growth factor (TGF)-β superfamily that signals to inhibit cell growth and studies with genetic siRNA knockdown of BMPR2 in human PAH endothelial cells demonstrate higher levels of glycolysis; healthy human endothelial cells transfected with mutant BMPR2 have greater glycolysis and increased expression of glycolytic mediator biosynthetic pathways [48,49]. Shared inheritance of particular mutations that encode genes that are important for metabolic regulation of glycolysis and intracellular bioenergetics are common between DM and PAH.

## 9. Shared Risk Factors and Clinical Implications

The identification of shared risk factors, such as obesity, diabetes, and dyslipidemia, underscores the link between insulin resistance and PH. Glucose intolerance and insulin resistance have been identified as novel risk factors in patients with pulmonary arterial hypertension [50]. Insights into the relationship between PH and insulin resistance have implications for patient management. Indeed, targeted approaches to address both metabolic and cardiovascular aspects of PH could prove more effective in improving outcomes for individuals with comorbid PH and insulin resistance. Individuals with diabetes may have an increased risk of developing pulmonary hypertension, and vice versa [51]. Indeed, a unique metabolomic endophenotype with abnormal glucose uptake in the right ventricle of patients could link altered glucose metabolism to pulmonary hypertension severity and outcomes [52]. Management strategies need to address related clinical factors in order to comprehensively to improve overall outcomes. These modifiable clinical factors include some of the following.

### 9.1. Hypoxia

Chronic and acute hypoxemia promotes pulmonary vasoconstriction as an initial compensatory physiologic phenomenon in order shunt blood flow to intact lung parenchyma in order to maximize efficiency of gas exchange (oxygen and carbon dioxide). This physiologic concept of hypoxic pulmonary vasoconstriction can become pathologic in the context of chronic unopposed hypoxemia. In PH, chronic vascular remodeling leads to narrowing of the pulmonary vasculature preferentially affecting the distal smaller arterioles and compromising blood flow to the alveolar-capillary unit. Similarly, impaired gas exchange and chronic tissue hypoxia can contribute to tissue ischemia and insulin resistance across microvascular beds throughout the body, leading to impaired glucose metabolism [53]. Hypoxemia is associated with an increase in sympathetic activity, which is a well-known pathophysiological mechanism in vascular diseases and insulin resistance. Chronic and intermittent hypoxia associated with obstructive sleep apnea (see below) lead to attenuated insulin secretion in pancreatic β cells [53]. Among the molecular mechanisms that likely promote the shift to glycolysis and away from oxidative glucose metabolism in PAH is the pathologic accumulation of hypoxia-inducible factor (HIF)-1α. HIF-1α is a transcription factor that plays a role in a variety of cellular functions, including proliferation, angiogenesis, survival, and metabolism [53].

### 9.2. Obesity and Obstructive Sleep Apnea

Obesity is an expanding worldwide public health challenge with rising prevalence, and is predicted to contribute to the increasing burden of cardiovascular disease, diabetes and cancer. Mechanisms associating obesity to PAH are not well understood; however, both conditions share several underlying pathological mechanisms. The metabolic changes that arise in obese individuals may contribute to the development of a pathophysiological environment that facilitates pulmonary vascular remodeling contributing to the development and/or exacerbation of PAH [54]. PAH and obese patients share comorbidities and 30% to 40% of patients with PAH are reported as obese [54]. Obesity, a common risk factor for insulin resistance and metabolic syndrome, is also associated with an increased risk of pulmonary hypertension through higher risk of developing obstructive sleep apnea, a type of sleep-disordered breathing and hypoventilation which may promote pulmonary vasculopathy and is classified as a causal factor for the WHO Group 3 PH. Intermittent hypoxemia during sleep increases selenoprotein P, which is one of the hepatokines, as well as TNF-α, CCL-2, and resistin, members of adipokines, to induce insulin resistance via direct cellular mechanisms [54]. Shared risk factors may contribute to the co-occurrence of these conditions.

#### Obesity and Inflammation

Adipose tissue accumulation and dysfunction in obesity can result in chronic low-grade inflammation, insulin resistance may contribute to the development of PAH. Given the growing levels of obesity and the pivotal role of adipose tissue in metabolic health and disease, adipose tissue has potential as a direct or indirect therapeutic target in the treatment of obesity-associated PAH [55,56,57]. Modulation of sex steroids may be of particular benefit in the treatment of PAH, given the role of adipose tissue in exogenous estrogen production. In particular, obese PAH patients may respond well to any novel therapies that reduce the influence of estrogens. Drugs that inhibit estrogen synthesis and attenuate estrogen effects, including aromatase inhibitors (already broadly utilized in estrogen-receptor positive breast cancers), are now in clinical trials for PAH [58]. Genetic variations in estrogen signaling have been associated with PAH [59]. In one placebo-controlled randomized clinical trial of the aromatase inhibitor, anastrozole showed a reduction in circulating estrogen levels and a significant increase in functional status as measured by 6 min walk distance (6MWD) over a 12-week study period, along with a demonstrated decrease in estrogen levels [58]. Overall, the drug was safe and well-tolerated in this small “proof-of-principle” study. Increased estrogen receptor expression has also been observed in vascular smooth muscle cells from both male and female PAH patients [59]; administration of fulvestrant, another estrogen inhibitor used in breast cancer, decreases the expression of estrogen receptors and resulted in a higher 6MWD, increased stroke volume in postmenopausal women with PAH [60]. Combined administration of fulvestrant and anastrozole has also been shown to result in a marked improvement in hemodynamics and pulmonary vascular remodeling in a transgenic mouse model of PAH [61].

## 10. Conclusions

Pulmonary hypertension and hyperglycemia with associated insulin resistance share common pathological pathways, suggesting a potential interconnection. While the precise mechanisms linking these conditions require further investigation, recognizing and addressing the coexistence of pulmonary hypertension and metabolic disorders is essential for effective patient care. Understanding these intricate relationships is crucial for developing comprehensive management strategies that address both pulmonary hypertension and metabolic disorders in affected individuals. Interdisciplinary approaches, involving the combination of expertise from both cardiovascular and metabolic specialists, lifestyle modifications, pharmacological agents, and emerging therapies offer promise in addressing the complex interplay between insulin resistance and pulmonary hypertension. Recognizing the shared molecular mechanisms and risk factors provides a foundation for developing integrated therapeutic strategies that target both conditions simultaneously, potentially improving overall patient outcomes. Further research in large-scale cardiovascular registries is essential to unravel the complexities of this interplay and refine treatment modalities for individuals facing the challenges of comorbid pulmonary hypertension and insulin resistance.

## Figures and Tables

**Table 1:** Common inflammatory mediators between hyperglycemia and pulmonary hypertension.

Inflammatory Mediator	Relation to Hyperglycemia	Role in PH
TNF-α	Increased levels lead to impaired glucose utilization and insulin resistance TNF-α highly expressed in adipose tissue Normally inhibited by CD33 which is suppressed by hyperglycemia	Suppression of prostacyclin Decreased activity of pyruvate dehydrogenase leads to impaired apoptosis regulation in PASMCs
IL-6	Levels tend to correlate with HbA1c Circulating levels higher in individuals with insulin resistance and overt DM Administration of recombinant IL-6 has dose dependent increase in serum glucose	Levels tend to correlate to disease severity Higher expression seen in cultured PASMCs from PAH patients
CRP	Serum levels correlate with development of T2DM Mice fed high-fat diets have CRP overexpression leading to insulin resistance	Levels are an independent risk factor for CV disease Leads to expression of adhesion molecules on vascular endothelium Higher levels portend worse outcomes and poor response to therapy
NF-kB	Implicated in development of insulin resistance Activated by AGEs in vascular smooth muscle cells which formed in the face of prolonged hyperglycemia	Pathway is activated within plexiform lesions from PAH patients Inhibition reduces luminal obliteration
MCP-1	Elevated in both T1DM and T2DM Increased expression in response to hyperglycemia	Plasma levels 2x higher in iPAH patients compared to controls Leads to proliferation of PASMCs
